# Effect of short-term exposure to high temperatures on the reproductive behavior and physiological enzyme activities in the fruit fly *Zeugodacus tau* (Walker)

**DOI:** 10.3389/fphys.2023.1036397

**Published:** 2023-02-10

**Authors:** Mao Li, Xiao-Man Wei, Juan Li, Shi-Ming Wei, Jin-Long Zhang, Guo-Hua Chen, Xiao-Ming Zhang

**Affiliations:** College of Plant Protection, Yunnan Agricultural University, Kunming, China

**Keywords:** *Zeugodacus tau*, short-term high temperatures, mating behavior, oviposition, physiological enzyme

## Abstract

*Zeugodacus tau* is an economically important invasive pest of various vegetables and fruits. In this study, we evaluated the effects of short-term (12 h) exposure to high temperatures on the reproductive behaviors and physiological enzyme activities of adult *Z. tau* flies. When compared to the control group, the mating rate in the treated group increased significantly after exposure to 34°C and 38°C. After 34°C exposure, the mating rate of the control♀-treated♂ mating was the highest (60.0%). The use of high temperatures for a short period reduced the pre-mating period and lengthened the duration of copulation. After 38°C exposure, the mating between treated♀ and treated♂ had the shortest pre-mating period of 39.0 min and the longest copulation duration of 67.8 min. Mating after a brief exposure to high temperatures had a negative impact on female reproduction, whereas mating with males who previously had a brief exposure to 34°C and 38°C significantly increased female fecundity. After 40 °C exposure, the mating between treated♀ and control♂ showed the lowest fecundity and hatching rate of 293.25 eggs and 25.71%, respectively. The mating between control♀ and treated♂ showed the highest fecundity of 1,016.75 eggs after exposure to 38°C. The SOD, POD, and CAT activities exhibited significant changes (increase or decrease) after the short-term exposure of *Z. tau* adults to high temperatures. After being exposed to 38°C, SOD activity increased by 2.64 and 2.10 times in females and males in the treated group, respectively, compared to the SOD activity in the control group. The AchE, CarE, and GST activities first increased and then decreased with the increase in temperature. CarE activity changed the most after exposure to 38°C, with females and males in the treated group increasing by 7.81 and 1.69 times, respectively, compared to the activity in the control group. In conclusion, mating strategy and physiological stress are important adaptive mechanisms of *Z. tau* for adapting to short-term heat stress in a sex-specific manner.

## 1 Introduction

The fruit fly *Z. tau* Walker (Diptera: Tephritidae) is an economically important agricultural pest of various fruits and vegetables. They are widely distributed in Southeast Asia and the South Pacific regions (Jaleel et al., 2018). It is classified as a complex species consisting of nine sibling species (*Zeugodacus tau* A, B, C, D, E, F, G, I, and J) (Baimai et al., 2000; Zaelor and Kitthawee, 2018). The females of *Z. tau* lay eggs beneath the skin of fruits and cause irreversible damage. Additionally, *Z. tau* larvae exhibit an innate feeding behavior of tunneling into the core of fruits, which allows them to escape insecticides (Zheng and Wei, 2019; Canhanga et al., 2020). Temperature is a critical factor in limiting the distribution of ectotherms (Santana et al., 2019). The abnormal changes in climate caused by global warming have a negative impact on pest survival and reproduction (García-Robledo et al., 2016; Ma et al., 2021). Therefore, understanding the impact of short-term high-temperature episodes on *Z. tau* is critical.

High-temperature and low-temperature stress irreversibly damages the function and structure of the reproductive system, thus, affecting the mating behavior of ectotherms (Singh et al., 2015; Enos and Kozak, 2021). High and low temperatures affect the mating behavior of fruit flies, including the mating-related traits of mating latency, duration of copulation, mating frequency, the number of progeny produced, *etc.* (Dev et al., 2013; Stazione et al., 2019; Singh et al., 2022). For example, keeping individuals of *Z. cucurbitae* for 1 h at 45 °C significantly stimulated the mating of *Zeugodacus cucurbitae* (Zeng et al., 2018). Females of *Drosophila melanogaster* recovered faster from cold shock in terms of mating latency, mating success, and progeny production; males recovered faster in terms of mating latency, fertility, sperm competitive ability, and progeny production (Singh and Prasad, 2016; Singh et al., 2017). At 25°C, however, *A. ipsilon* had the highest mating percentage. Calling and mating behaviors were prevent in females of *Agrotis ipsilon* at temperatures lower or higher than 25°C (Xiang et al., 2018). This effect of high and low temperatures on the reproductive behavior of ectotherms altered reproductive fitness. This alteration manifested mainly as a delay in the pre-oviposition and peak period of oviposition, a reduction in egg production and hatching rate, a gradual shortening of the life span in both males and females, and an increase in the female-to-male offspring ratio (Zhou et al., 2011; Stazione et al., 2021). However, the evolution of reproductive traits was found to have no apparent life-history associated cost for cold shock resistance (Singh et al., 2021). Huang et al. (2020) reported that short-term exposure to temperatures above 42°C was unsuitable for the development of *Z. tau* individuals, while short-term treatment with temperatures above 40°C was unsuitable for reproduction in *Z. tau*. However, more research on the reproductive behavior of *Z. tau* under short-term high-temperature exposure is needed.

Under high temperatures, several stress reactions occur in ectotherms. Under stress, ectotherms accumulate toxic metabolites and generate high levels of free radicals. To overcome this problem, protective enzyme systems, including antioxidant and detoxification systems, have evolved in ectotherms (Guo et al., 2018; Shankarganesh et al., 2022). Superoxide dismutase (SOD), catalase (CAT), and peroxidase (POD) are the three main antioxidant enzymes found in ectotherms (Cai et al., 2018). The three main detoxification enzymes in ectotherms are acetylcholinesterase (AchE), carboxylesterase (CarE), and glutathione S-transferase (GST) (Li et al., 2022). Abiotic stresses can have an impact on both enzyme activity and insect reproduction (Roma et al., 2021; Cai et al., 2022). According to previous research, elevated CO_2_ affects the population development of thrips as well as the activities of detoxification enzymes. The fecundity of *Frankliniella occidentalis* and *Thrips hawaiiensis* increased and decreased significantly as CO_2_ concentration increased. This could be due to the activity of detoxifying enzymes (Cao et al., 2020). The effects of sodium fluoride (NaF) on the reproduction and antioxidant enzyme activities of *Bombyx mori* larvae revealed that NaF in mulberry leaves fed to *B. mori* larvae would cause reproductive damage, a disorder of the antioxidant system in the gonads, and oxidative stress (Tang et al., 2016). External stress-induced oxidative stress may be an important factor in determining their toxicity to insects (Paweł and Grzegorz, 2021). There is, however, little direct evidence of a link between insect enzyme activity and reproduction. In other organisms, particularly marine organisms, oxidative stress caused by abiotic stresses has been linked to impaired reproduction (Han et al., 2014). According to recent research, harmful environments may cause reproduction toxicity by disrupting the expression of reproduction and detoxification-related genes and inducing oxidative stress in organisms (Liu et al., 2022).

The courtship and mating of *Z. tau* occur mainly in the evening (Shamshir and Wee, 2019). Although global warming is characterized primarily by an increase in daytime temperatures (Ma et al., 2021), exposure to such high-temperature conditions affects the subsequent mating activities of the individuals of *Z. tau* in the evening. Few studies have investigated the effect of temperature on the behavior of fruit flies concerning finding a mate and coordinating during mating (Singh et al., 2015; Singh et al., 2021; Singh et al., 2022). Furthermore, male and female behavior and physiology differ, particularly in terms of thermal sensitivity and physiological enzyme activities (Brandt et al., 2018; Macchiano et al., 2019; Singh et al., 2022). Therefore, at higher temperatures, females and males may exhibit behavioral mismatches when seeking or being receptive to potential mates (Singh and Prasad, 2016; Singh et al., 2017; Leith et al., 2020). In this context, we hypothesized that *Z. tau* males and females would use different mating strategies and physiological response mechanisms to adapt to high-temperature stress after a brief high-temperature exposure, and that mating immediately following a brief high-temperature exposure would affect subsequent oviposition behavior at 25°C. The experiment was carried out in an artificial climate chamber, and the mating behaviors of the animals on the same day were evaluated after they were exposed to high temperatures for a short period during the day and then returned to 25°C at dusk. The mating rate, pre-mating period, duration of copulation, and number of offspring produced by mating were all assessed. The activities of antioxidant and detoxification enzymes were determined immediately after exposing individuals of *Z*. *tau* to high temperatures for a short period of time. By conducting these experiments, we investigated the adaptation of mating behavior and the mechanism of physiological enzyme regulation in *Z. tau* after short-term exposure to high temperatures. The findings of this study could provide a theoretical foundation for the comprehensive management of *Z. tau* in future climate scenarios caused by global warming.

## 2 Materials and methods

### 2.1 Rearing and temperature settings

Individuals of *Z. tau* were collected during 2021 from a field of *Cucurbita pepo* in Mengzi City (103.24°E, 23.30°N), Yunnan Province, South China. The study area has an annual average temperature of 17°C–26°C, and extreme summer heat waves (≥34°C) occur in Mengzi City (https://lishi.tianqi.com/mengzi/202207.html). We collected samples of *Z. tau* adults (*n* = 3–5) from the study area to confirm their identity. These individuals were tested in the laboratory using their mtCOI sequences (Kitthawee and Julsirikul, 2019). The results of the identification revealed that the *Z. tau* individuals belonged to *Z. tau* A. (the identity of the sample deposited in GenBank, no. OP735533). Under laboratory conditions of 25°C ± 1°C, 70% ± 5% RH, and a 14 h:10 h (light: dark) photoperiod, a stable experimental population of *Z. tau* was established. Adults and larvae were fed a synthetic diet (Huang et al., 2020). Individuals of *Z. tau* were reared for more five generations.

The experiments were conducted in an artificial climate chamber (model BIC-300, Shanghai Boxun Medical Biological Instrument Co., Ltd., China) at 25°C ± 1°C, 70% ± 5% RH, and 14 h: 10 h (light: dark) photoperiod (Abraham et al., 2016). Previous research has shown that keeping *Z. tau* adults at 34°C for 12 h does not effect on their survival; however, no individuals can survive for 12 h at 44°C (Huang et al., 2020). Therefore, the temperature exposure groups were evaluated 12 h after being exposed to 34, 36, 38, 40, 42, and 44 °C, while the control group was evaluated at 25°C.

### 2.2 Effects of short-term exposure to high temperatures on *Z. tau* adults’ survival

The adults that emerged within 24 h were selected. Male and female adults were separated and transferred to plastic cages for rearing (35 cm × 35 cm × 35 cm). The sexually mature virgin females and males were chosen after 30 days of rearing at 25°C, and each group of 300 females or males was considered one replicate. The adults were placed in an artificial climate chamber and subjected to temperature of 34, 36, 38, 40, 42, and 44°C for 12 h (light period). After 12 h, the number of dead *Z. tau* adults, survival rate, semi-lethal temperature (LT_50_), and lethal temperature were calculated. Each recorded temperature was then chosen as a treatment, and four such treatments were established, each with four replicates.

### 2.3 Effects of short-term exposure to high temperatures on *Z. tau* mating

The experiment was carried out at a temperature of 25°C, and the surviving adult females and males from the previous step were chosen as test insects. After 12 h of exposure at 34, 38, 40, and 42°C, the mating behavior was evaluated within 12 h at 25 °C on the same day because the survival rate test revealed no difference in adult survival at 34°C and 36°C. Three short-term high-temperature exposure conditions were used: a) females were exposed to short-term high-temperature stress before mating with unexposed males (treated♀-control♂); b) males were exposed to short-term high-temperature stress before mating with unexposed females (control♀-treated♂), and c) both females and males were exposed to short-term high-temperature stress before mating (treated♀-treated♂). The control group (control♀-control♂) were formed when females and males were mated at 25 °C without short-term high temperature exposure (control♀-control♂). To allow mating, each pair of female and male in the various combinations was placed inside a 180-mL disposable plastic cup (inverted). To form the base, a suitable cylinder of flower mud was cut out at the mouth of the cup. During the dark period, the disposable plastic cup containing the female-male pair was placed in the artificial climate box at 25 °C. Every 15 min, mating was observed. The mating rate, pre-mating period, and duration of copulation were calculated. A mating combination after exposure to different short-term high temperatures was treated as one treatment, with a total of four short-term high temperatures, three mating combinations, and forming 12 treatments. One replicate consisted of 30 pairs of adults, and four replicates were performed.

### 2.4 Effects of mating after short-term exposure to high temperatures on *Z. tau* oviposition

The experiments were carried out at a temperature of 25°C. The mated females in the previous step were chosen for the oviposition experiment, and each female was raised in a 180-mL disposable plastic cup. Cotton balls, artificial feed, and a piece of *C. pepo* (1 cm^3^) were placed in the plastic cup to provide water and food, as well as to aid in oviposition. All materials were replaced every 24 h. The fecundity and number of eggs laid by females were observed and recorded until they died. Four replicates were performed, each with 20 females.

### 2.5 Effects of short-term exposure to high temperatures on the physiological enzyme activities of *Z. tau* individuals

Since the survival rate test revealed that only a few adults survived at 42 °C, physiological enzyme activity was measured only at 34, 38, and 40 °C. Virgin females and males (30 days old) were chosen at random and exposed to one of the above temperatures for 12 h, while the control group was kept at 25 °C. The activities of SOD, POD, CAT, AchE, GST, and CarE were determined using the kit’s instructions (Beijing Solarbio Science & Technology Co., Ltd., Beijing, China). The enzyme activity was measured using UV-Vis spectrophotometry based on the sample mass (Zhang et al., 2020). Each treatment received three replicates.

### 2.6 Statistical analysis

Based on linear regression, the semi-lethal temperature (LT_50_), 95% confidence intervals, regression equation, and correlation coefficient for *Z. tau* were calculated. One-way ANOVA was used to evaluate the reproductive behaviors and activity tests of the physiological enzymes of *Z. tau*. The LSD multiple comparison method was used to compare significant differences between different mating combinations and between females and males at different temperatures. The SPSS software was used for all analyses (version 25.0; SPSS, Chicago, IL, United States). Origin software was used to draw the figures (version 9.1 pro; OriginLab, Northampton, MA, United States).

## 3 Results

### 3.1 The survival rate of *Z. tau* adults exposed to high temperatures for a short duration

The different short-term high-temperature treatments affected the survival rate of *Z. tau* adults. The survival rate of females and males was significantly lower after 38, 40, and 42 °C exposure than that at the control temperature (females: *F*_6,27_ = 398.1320, *p =* 0.0001; males: *F*_6,27_ = 168.5890, *p =* 0.0001). Male and female survival rates decreased as treatment temperature increased, and the female survival rate was higher than the male survival rate (Table 1). The LT_50_ value for the males was 38.46°C, which was 0.70 °C lower than that of females (Table 2).

**TABLE 1:** The survival rate of *Z. tau* adults exposed to different high-temperature treatments for 12 h.

Temperature (°C)	Females	Males
25	100.0 ± 0.0a	100.0 ± 0.0a
34	99.02 ± 0.98a	96.80 ± 1.62a
36	98.89 ± 0.11a	95.89 ± 0.48a
38	75.28 ± 4.31 b	58.91 ± 8.12 b
40	33.14 ± 3.95c	20.01 ± 3.49c
42	5.44 ± 0.72 d	5.16 ± 1.85 d
44	0.0 ± 0.0 d	0.0 ± 0.0 d

Data are presented as mean ± SE. The different lowercase letters within the same column indicate significant differences (*p* < 0.05).

**TABLE 2:** The LT_50_ values of *Z. tau* adults after 12 h of exposure to different high-temperature treatments.

	Regression equation	Correlation coefficient r)	LT50 (95% confidence interval)
Females	Y = 51.953 X-82.753	0.920	39.161 (38.544–39.774)
Males	Y = 44.891 X-71.155	0.912	38.464 (38.071–38.855)

### 3.2 Effect of different short-term high-temperature treatments on reproductive behaviors in *Z. tau*

#### 3.2.1 The mating rate of *Z. tau* adults exposed to different short-term high-temperature treatments

**FIGURE 1:**
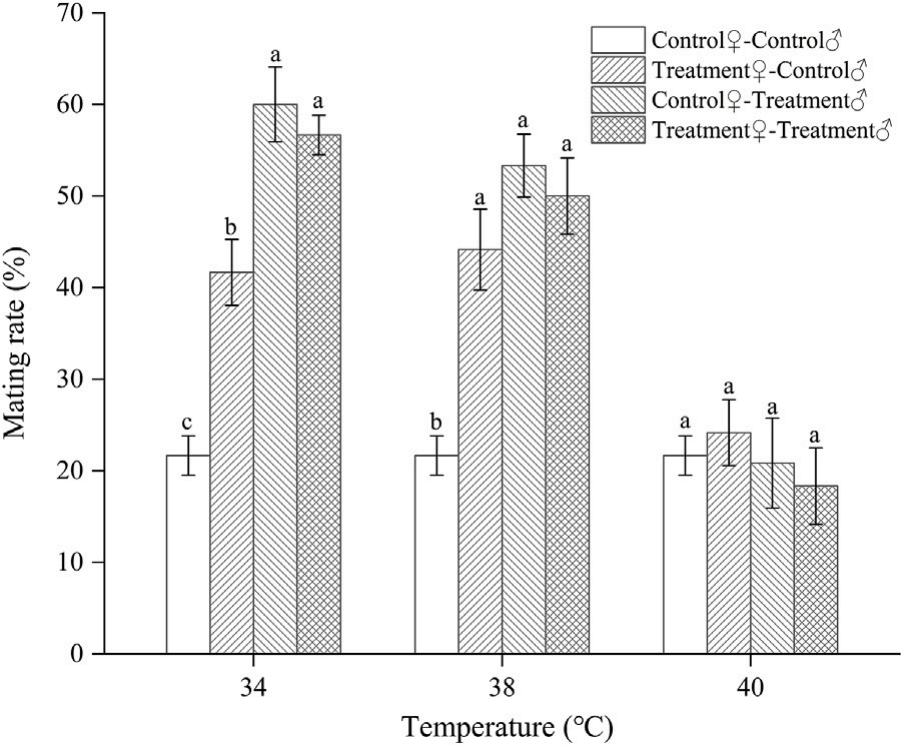
The mating rate of *Z. tau* individuals exposed to different high-temperature treatments for 12 h. Data are presented as the mean ± SE. Different lowercase letters above the bars indicate significant differences between different mating combinations at the same temperature (*p* < 0.05). No mating occurred within 24 h after the individuals were exposed to 42°C for 12 h.

Exposure to high temperatures for a short duration significantly affected the subsequent mating rate of *Z. tau* individuals that day. The mating rate of females was different from that of males after the same temperature exposure. The mating rate was significantly higher after 34 °C and 38 °C exposure compared to that at 25 °C (34 °C: *F*_3,15_ = 24.4930, *p =* 0.0001; 38 °C: *F*_3,15_ = 14.1660, *p =* 0.0003). After these two temperatures exposure, the control♀-treated♂ combination increased the mating rate by the most (60.0% and 53.25%, respectively), followed by the treated♀- treated♂ and treated♀-control♂ combinations. After short-term exposure to 40 °C, the mating rates of all mating combinations were lower than those of the control, except for the treated♀-control♂ combination (*F*_3,15_ = 0.4980, *p =* 0.6908). After short-term exposure to 42 °C, no mating occurred between males and females (Figure 1).

#### 3.2.2 The pre-mating period in *Z. tau* adults exposed to different short-term high-temperature treatments

The pre-mating period of *Z. tau* individuals was shortened after a brief exposure to high temperatures. The pre-mating period became shorter at first, then longer as the treatment temperature increased. For all mating combinations, the shortest pre-mating period occurred after 38 °C exposure (*F*_3,15_ = 9.290, *p =* 0.0001). The order was as follows: treated♀-treated♂ (39.0 min), treated♀-control♂ (66.47 min), control♀-treated♂ (78.59 min), and control♀-control♂ (123.40 min). Among all mating combinations, treated♀-treated♂ had the shortest pre-mating period in all treated groups (*F*_3,15_ = 8.3580, *p =* 0.0002) (Table 3).

**TABLE 3:** The pre-mating period of *Z. tau* adults exposed to different high-temperature treatments for 12 h.

Temperature (°C)	Pre-mating period (min)			
	Treated♀-control♂	Control♀-treated♂	Treated♀-treated♂	Control♀-control♂
34	97.27 ± 7.79ABab	109.67 ± 11.82ABab	85.0 ± 10.05BCb	123.40 ± 12.91a
38	66.47 ± 6.67Bb	78.59 ± 7.15Bb	39.0 ± 9.76Cc	123.40 ± 12.91a
40	98.75 ± 12.77ABa	100.0 ± 8.66ABa	93.75 ± 9.87ABa	123.40 ± 12.91a
25	123.40 ± 12.91A	123.40 ± 12.91A	123.40 ± 12.91A	—

Data are mean ± SE. the different lowercase letters after the same row data indicate significant differences between different mating combinations at the same temperature, and the different uppercase letters after the same column data indicate significant differences between the same mating combinations at different temperatures (*p* < 0.05). The same is below.

#### 3.2.3 Duration of copulation in *Z. tau* adults exposed to different short-term high-temperature treatments

The duration of copulation increased after treatment in *Z*. *tau* adults. Copulation duration was significantly longer after 38 °C exposure than in the control (*F*_3,15_ = 4.380, *p* = 0.0069). The longest durations of copulation for the treated♀-control♂ and treated♀-treated♂ combinations were 648.07 min and 678.0 min, respectively, both of which were treated after 38 °C exposure (treated♀-control♂: *F*_3,15_ = 2.3110, *p =* 0.0436; treated♀-treated♂: *F*_3,15_ = 4.8960, *p =* 0.0051). The longest duration of copulation for the control♀-treated♂ combination was 640.0 min which occurred after treatment after 34 °C exposure (*F*_3,15_ = 2.1810, *p =* 0.0981) (Table 4).

**TABLE 4:** The duration of copulation of *Z. tau* adults exposed to different short-term high-temperature treatments for 12 h.

Temperature (°C)	Duration of copulation (min)			
	Treated♀-control♂	Control♀-treated♂	Treated♀-treated♂	Control♀-control♂
34	627.42 ± 16.87ABa	640.0 ± 18.43Aa	622.47 ± 11.29Ba	595.47 ± 16.23a
38	648.07 ± 14.82Aa	637.81 ± 9.03Aa	678.0 ± 18.67Aa	595.47 ± 16.23b
40	609.50 ± 12.75ABa	626.0 ± 14.33Aa	599.83 ± 15.95Ba	595.47 ± 16.23a
25	595.47 ± 16.23B	595.47 ± 16.23A	595.47 ± 16.23B	—

#### 3.2.4 Fecundity of *Z. tau* adults exposed to different short-term high-temperature treatments

Mating of *Z. tau* adults after short-term high-temperature exposure affected subsequent fecundity. After 34 °C and 38 °C exposure, the fecundity of control♀-treated♂ was the highest, with the values of 1,004.11 eggs and 1,016.75 eggs, respectively (34 °C: *F*_3,15_ = 16.0720, *p =* 0.0001; 38 °C: *F*_3,15_ = 5.6660, *p =* 0.0044). After 40 °C exposure, fecundity decreased significantly (*F*_3,15_ = 6.3760, *p =* 0.0036). The fecundity of treated♀-control♂ reached the minimum level of 293.25 eggs after 40 °C exposure (*F*_3,15_ = 3.3450, *p =* 0.0345) (Table 5).

**TABLE 5:** Fecundity of *Z. tau* adults exposed to different high-temperature treatments for 12 h.

Temperature (°C)	Fecundity per females			
	Treated♀-control♂	Control♀-treated♂	Treated♀-treated♂	Control♀-control♂
34	411.25 ± 62.97ABb	1,004.11 ± 77.80Aa	541.33 ± 69.62Ab	545.29 ± 35.48b
38	385.80 ± 38.80Bb	1,016.75 ± 116.53Aa	538.60 ± 41.84Ab	545.29 ± 35.48b
40	293.25 ± 45.62Bb	373.50 ± 52.70Bb	299.50 ± 55.98Bb	545.29 ± 35.48a
25	545.29 ± 35.48A	545.29 ± 35.48B	545.29 ± 35.48A	—

#### 3.2.5 Hatching rate of the eggs of *Z. tau* individuals exposed to different short-term high-temperature treatments

Mating of *Z. tau* adults after short-term high-temperature exposure affected subsequent hatching rate of eggs. The hatching rate decreased to different degrees in different treated groups, among which the hatching rate of the eggs of treated♀-control♂ was the lowest (34 °C: *F*_3,15_ = 3.0090, *p =* 0.0456; 38 °C: *F*_3,15_ = 0.9680, *p =* 0.4205; 40 °C: *F*_3,15_ = 9.9470, *p =* 0.0003). The lowest hatching rate of the eggs of treated♀-control♂ was only 25.71% after 40 °C exposure (*F*_3,15_ = 7.0480, *p =* 0.0010). The hatching rate of control♀-treated♂ eggs increased with treatment temperature, reaching a maximum of 82.25% after 40 °C exposure, which was close to the control (*F*_3,15_ = 2.8630, *p* = 0.0453). The hatching rate of treated♀-treated♂ eggs decreased with increasing treatment temperature (*F*_3,15_ = 1.1530, *p* = 0.3491). (Table 6).

**TABLE 6:** The hatching rate of the eggs of *Z. tau* adults exposed to different high-temperature treatments for 12 h.

Temperature (°C)	Hatching rate (%)			
	Treated♀-control♂	Control♀-treated♂	Treated♀-treated♂	Control♀-control♂
34	58.07 ± 7.59Bb	61.62 ± 5.19Bb	72.09 ± 5.86Aab	82.98 ± 3.77a
38	68.26 ± 6.27ABa	74.05 ± 7.49Aba	68.99 ± 4.41Aa	82.98 ± 3.77a
40	25.71 ± 2.97Cb	82.25 ± 5.21Aa	65.78 ± 9.92Aa	82.98 ± 3.77a
25	82.98 ± 3.77A	82.98 ± 3.77A	82.98 ± 3.77A	—

### 3.3 Effect of short-term exposure to high temperatures on the physiological enzyme activities of *Z. tau* individuals

#### 3.3.1 Antioxidant enzyme activities in *Z. tau* individuals exposed to different short-term high-temperature treatments

**FIGURE 2:**
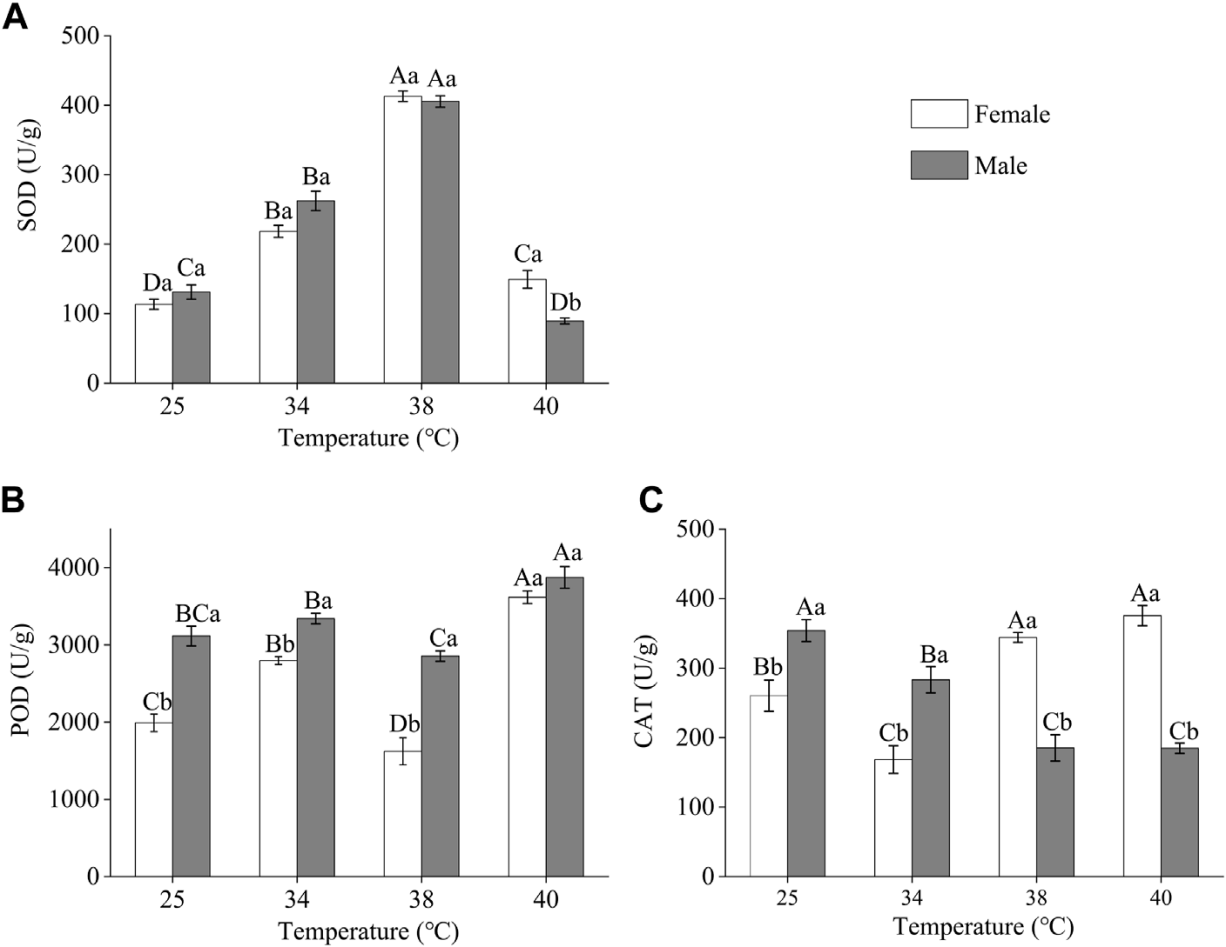
Antioxidant enzyme activities in *Z. tau* adults exposed to different high-temperature treatments for 12 h. Data are presented as the mean ± SE. Different lowercase letters above bars represent a significant difference in the antioxidant enzyme activity between females and males at the same temperature. In contrast, different uppercase letters above bars indicate a significant difference in the antioxidant enzyme activities of females/males at different temperatures (*p* < 0.05) **(A)** SOD **(B)** POD, and **(C)** CAT.

The antioxidant enzyme activities of *Z. tau* individuals were affected by short-term exposure to high temperatures, and the enzyme activities of females and males differed (Figure 2). The SOD activity of both females and males increased and then decreased as the treatment temperature increased. The highest SOD activity was observed after 38°C exposure, SOD activity increased by 2.64 and 2.10 times in females and males in the treated group, respectively, compared to the SOD activity in the control group (females: *F*_3.11_ = 202.3730, *p =* 0.0001; males: *F*_3.11_ = 210.920, *p =* 0.0001). After 40 °C exposure, the SOD activity in males was lower than that in the individuals in the control group and significantly lower than that in females (*F*_1,5_ = 19.6660, *p =* 0.0114) (Figure 2A). The highest POD activity was observed after 40 °C exposure. After this temperature exposure, the POD activity of females and males increased by 0.88 times and 0.24 times, respectively, compared to the activity in the control group (females: *F*_3,11_ = 56.4710, *p =* 0.0001; males: *F*_3,11_ = 16.7520, *p =* 0.0008) (Figure 2B). The CAT activity in females was the highest after 40 °C exposure, and after this temperature exposure, the activity increased by 0.44 times compared to the control (*F*_3,11_ = 27.2690, *p =* 0.0001). In males, CAT activity decreased as treatment temperature increased (*F*_3,11_ = 18.2520, *p =* 0.0001) (Figure 2C).

#### 3.3.2 Activities of detoxifying enzymes in *Z. tau* individuals exposed to different short-term high-temperature treatments

**FIGURE 3:**
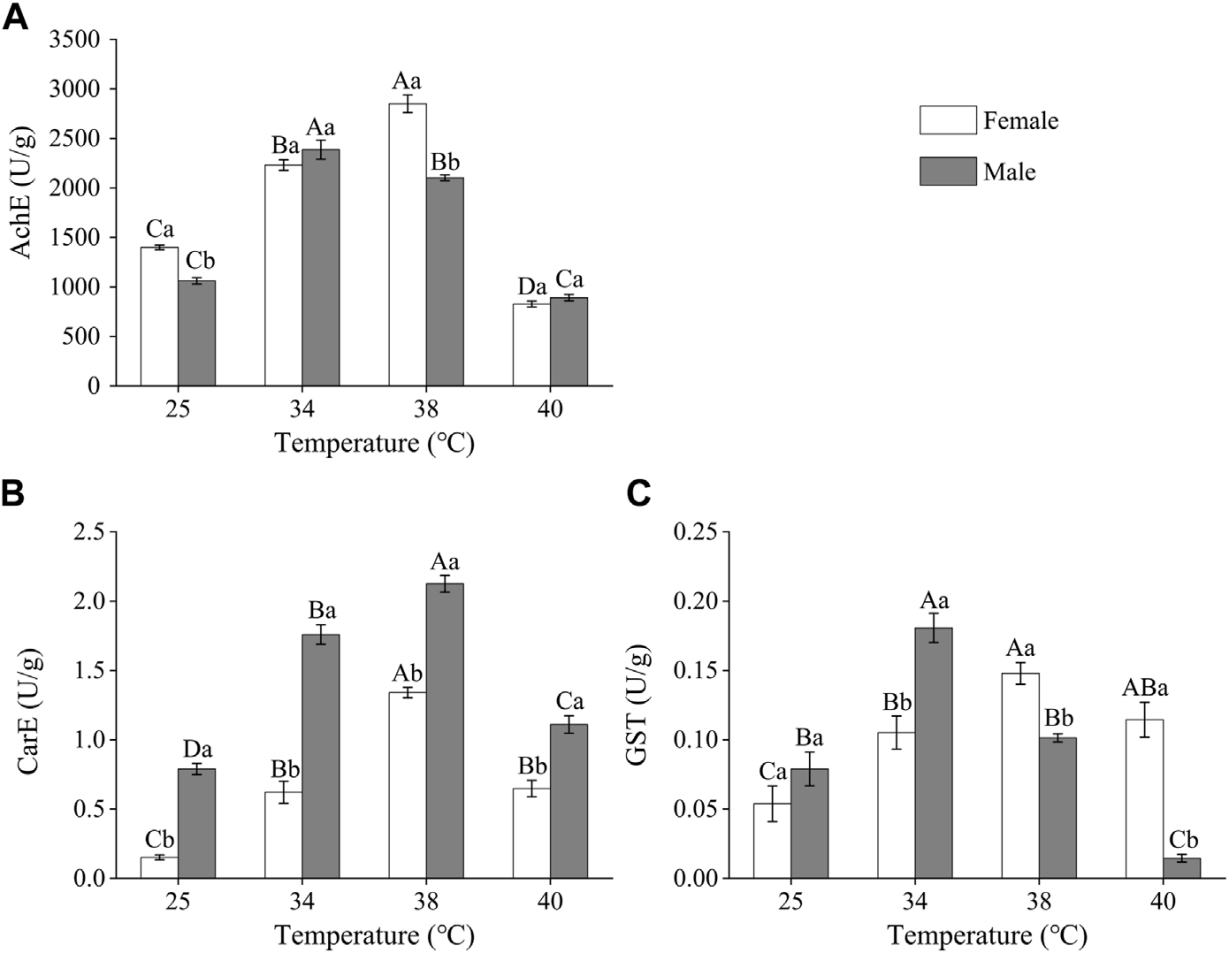
The activities of detoxifying enzymes in *Z. tau* adults exposed to different high-temperature treatments for 12 h. Data are presented as the mean ± SE. Different lowercase letters above bars represent a significant difference in the detoxifying enzyme activity between females and males at the same temperature, while different uppercase letters above bars indicate a significant difference in the detoxifying enzyme activities of females/males at different temperatures (*p* < 0.05) **(A)** AchE **(B)** CarE, and **(C)** GST.

As the treatment temperature increased, the activity of the detoxifying enzymes in *Z. tau* individuals first increased and then decreased, with differences between females and males (Figure 3). The AchE activity of females and males was the highest after 38°C and 34°C exposure, respectively. The activities in females and males increased by 1.04 and 1.25 times, respectively, when compared to the control group (females: *F*_3,15_ = 161.3250, *p* = 0.0001; males: *F*_3.15_ = 181.6750, *p* = 0.0001). (Figure 3A). CarE activity was higher in the short-term high-temperature treated groups than in the control group, with the highest activity recorded after 38°C exposure. Females’ and males’ CarE activities after 38 °C exposure increased 7.81 times and 1.69 times, respectively, compared to the control group (females: *F*_3.11_ = 83.3130, *p* = 0.0010; males: *F*_3,11_ = 101.2170, *p* = 0.0001) (Figure 3B). The GST activity in females and males was the highest after 38 °C and 34 °C, respectively. After these temperatures exposure, the GST activity in females and males increased by 1.75 times and 1.29 times, respectively, compared to the activities in the control group (females: *F*_3,15_ = 8.7750, *p =* 0.0038; males: *F*_3,15_ = 14.4580, *p =* 0.0014). The GST activity in males was higher than that in females after 25°C and 34°C exposure, while the activity in females was higher than that in males after 38°C and 40°C exposure (25°C: *F*_1,5_ = 0.9160, *p =* 0.3926; 34°C: *F*_1,7_ = 8.6110, *p =* 0.0261; 38°C: *F*_1,7_ = 30.9230, *p =* 0.0051; 40°C: *F*_1,7_ = 59.9920, *p =* 0.0015) (Figure 3C).

## 4 Discussion

This study’s findings highlighted three key points. First, mating of *Z. tau* adults was promoted after short-term high-temperature exposure after 34°C and 38°C exposure. In addition, the pre-mating period was shorter, and copulation lasted longer. Second, mating of *Z. tau* adults after short-term high-temperature exposure affected subsequent oviposition. Female fecundity and hatching rate decreased after short-term high-temperature stress. In contrast, mating with males who previously had a brief exposure to 34°C and 38°C significantly increased female fecundity. Third, short-term exposure to high temperatures affected the physiological metabolism of *Z. tau* adults; the individuals responded to this stress using their antioxidant and detoxification systems.

We discovered that short-term high-temperature treatment affected mating rate in *Z. tau*. Temperature changes in the environment can influence fruit fly mating behavior (Singh et al., 2015; Singh et al., 2021; Singh et al., 2022). Behavioral adaptations in ectotherms can help them to cope with high-temperature stress (Lachenicht et al., 2010). In this study, short-term exposure to high temperatures negatively affected the survival of *Z. tau* adults, although their mating rate increased after 34°C and 38°C exposure. Therefore, we inferred that changes in the mating behavior might be a survival strategy adopted by *Z. tau* adults to cope with high-temperature stress. Other organisms have shown similar behavioral adaptation strategies for survival under environmental stress, such as hormesis and stimulated parasitic behavior in *Encarsia formosa* after exposure to sublethal concentrations of spirotetramat (Yang et al., 2021), an increase in courtship and copulation behavior in *Trichogramma chilonis* exposed to an insecticide (Wang et al., 2018), and so on. Temperature also influences the expression of many behavioral traits in ectotherms, including several traits involved in pair formation and mating (Singh and Prasad, 2016; Singh et al., 2017; Leith et al., 2020). Adult males exhibit a series of courtship behaviors to attract females during fruit fly mating, while a large proportion of adult females refuse to mate (Shelly, 2001; Aquino and Joachim-Bravo, 2014). In this study, the treated♀-control♂ and control♀-treated♂ combinations of *Z. tau* exhibited significantly higher mating after 34°C and 38°C exposure compared to the individuals in the control group. This finding indicated that even short-term exposure to high-temperature stress within a certain temperature range can weaken sexual selection in females, but the courtship behavior of males may not be adversely affected. Whether external stimuli can regulate the courtship behavior in fruit flies is not known. However, environmental factors and the signaling behavior of males might be involved in the regulation of courtship behavior in fruit flies (Díaz-Fleischer and Arredondo, 2011).

Short-term exposure to high-temperature stress shortened the pre-mating period and prolonged the duration of copulation in *Z. tau*. Most studies have suggested that the pre-mating period of adults is the shortest at suitable temperatures, while the duration of copulation is shortened significantly with an increase in temperature (Katsuki and Miyatake, 2008; Yang et al., 2015). In this study, we discovered disparate results. These differences occurred most likely because previous studies were conducted at constant temperatures. In mating behavior, the duration of copulation is frequently regarded as important for sex selection and has a significant impact on the adaptability of adult males and females (Linn et al., 2007; De-Lima et al., 2021). In this study, the prolonged duration of mating after exposure to short-term high-temperature treatment might be related to the mating characteristics of *Z. tau*. Fruit flies demonstrate distinct circadian rhythms during mating behavior (Fletcher, 1987). Unlike many species that mate several times a day, individuals of *Z. tau* mate only once a day. Furthermore, despite the fact that *Z*. *tau* females mate with multiple males, they exhibit cyclic receptivity, which means that after each mating event, females resist mating for a period of time before regaining the ability to accept mates (Chinajariyawong et al., 2009). Therefore, when mating after short periods of heat stress, *Z. tau* may prefer to extend the mating time to ensure reproductive success.

Aside from directly causing mortality, high temperatures may have an indirect effect on population development by reducing reproductive adaptability (Tao et al., 2018; Zhou et al., 2020). This study looked into the oviposition process of *Z. tau* and discovered that short-term exposure to high-temperature treatment affected fecundity and hatching rate after mating. After short-term high-temperature treatment, fecundity and the hatching rate decreased in females, which was consistent with the finding of previous studies (Liang et al., 2021). High ambient temperatures adversely affect pest reproduction, although, in most studies, this conclusion was based on the results of high-temperature treatment of females or the simultaneous treatment of females and males (Zhou et al., 2020; Takeda, 2022). In this study, the fecundity of control♀-treated♂ increased significantly after 34°C and 38°C exposure, while the hatching rate increased with the increase in treatment temperature. Heat stress affects reproduction in adult males, and this has also been demonstrated in other species. For example, female *Bicyclus anynana,* after mating with heat-stressed males exhibited an increase in early fertilization (Janowitz and Fischer, 2011). Another study reported that short-term exposure to high temperatures was detrimental to the reproduction of male *Grapholita molesta* (Bao et al., 2019). This finding was different from the results of our study. Our findings on oviposition in the control♀-treated♂ combination, combined with the results of prolonged copulation, suggested that short-term exposure to high-temperature treatment significantly affected the fecundity and fertility of *Z. tau* males. This indicated an increase in the investment of adult males in subsequent reproduction following high-temperature stress. According to some studies, the presence of sperm in the reproductive tract of females can directly stimulate oviposition and increase fertility (Gromko et al., 1984; Tom and Nina 1998). Therefore, we speculated that the heat-stressed males produced greater quantities of sperm, which was then delivered to the females. These females consequently exhibited higher fecundity and hatching rate. In some studies, mating negatively affected females; even the secretions from male appendages reduced the number of eggs laid by the females and shortened their lifespan (Chapman et al., 1995; Siva-Jothy et al., 1998). In conclusion, mating of *Z. tau* adults after short-term exposure to high temperatures may be both advantageous and disadvantageous to their reproductive fitness.

The response of antioxidant enzymes to stress is an important strategy adopted by fruit flies to cope with temperature-related stress (Jia et al., 2011). In this study, the activities of the SOD, POD, and CAT enzymes in *Z. tau* adults were altered to varying degrees under short-term high-temperature treatment, indicating oxidative stress and physical damage to *Z. tau* adults due to high-temperature stress. After high-temperature treatment, these three antioxidant enzymes, particularly SOD, showed significant changes. SOD catalyzes the oxidation of superoxide to produce hydrogen peroxide (H_2_O_2_) (Larisa et al., 2021). It is an important enzyme that helps organisms resist oxidative stress. SOD activity increased significantly after 34 °C and 38 °C exposure in our study. Therefore, we inferred that high-temperature stress induced SOD activity in *Z. tau* adults, which led to effective scavenging of the oxygen free radicals and protected them from the damage caused by these reactive oxygen species. However, when the treatment temperature increased to 40°C, the high-temperature stress was beyond the tolerance range of *Z. tau* adults and the SOD activity decreased. These findings matched those of other studies on *Empoasca onukii* and *Frankliniella occidentalis* (Qiao et al., 2015; Yuan et al., 2021). After 40 °C exposure, the activity of oxidoreductase was higher in females than in males, implying that the oxidoreductase system was more efficient in females. This could be one of the reasons why males die at a higher rate than females under short-term high-temperature stress.

In fruit flies, the action of the detoxification enzyme system is important for coping with various environmental pressures (Wang et al., 2016). In this study, the activities of the three detoxifying enzymes AchE, CarE, and GST in *Z. tau* first increased and then decreased with the increase in the treatment temperature. The main factors associated with an increase in the activity of detoxification enzymes may include oxidative stress and increased metabolic waste at high temperatures. The variation in the activity of the detoxifying enzymes was similar to that reported in a study on *Tetrarcychus urticae* exposed to high-temperature stress (Wang et al., 2021). The differences in the changes in detoxification enzymes and their levels in male and female flies exposed to the same high temperature reflected the pest’s sex-specific adaptive capability. Short-term high temperatures were found to interfere with the behavioral and physiological processes of *Z. tau*, and it is speculated that metabolic differences between males and females may be one of the important factors causing the behavioral differences. There is little information on the relationship between enzyme activity and behavior in invertebrates. AChE activity can be used as a biomarker to evaluate changes in neurotoxicity and can affect related physiological and behavioral processes (Schweizer et al., 2019). The relationships between AChE inhibition and impairment of physiological and behavioral processes, particularly in vertebrate species, have been well explored (Bonansea et al., 2016; Pour et al., 2022). In *Gammarus fossarum*, for example, AChE inhibition impairs both feeding and locomotor behavior (Xuereb et al., 2009). In this study, some short-term high temperatures did increase AChE activity in *Z. tau* and changed its reproductive behavior. The persistent response of AChE may be the main mechanism of mating in *Z. tau* after short-term high temperature exposure. This study, however, does not confirm that there is a robust relationship between behavioral changes and AChE activity in *Z. tau*, and more research is needed to determine the quantitative relationship between them.

The findings of this study confirmed that the in response to short-term high-temperature environmental stress, the mechanisms of mating behavior, antioxidant defense, and detoxifying enzymes in *Z. tau* alter to cope with temperature changes and high-temperature stress, and that these mechanisms are sex-specific. Our findings may have important ecological implications for the reproduction and survival of *Z. tau* under global warming scenarios, and they provide a theoretical basis for predicting the population and range of distribution of this species under climate variability. We only evaluated and analyzed the mating process and the associated changes in *Z. tau* adults after short-term exposure to high-temperature conditions in this study; however, the mechanism underlying the regulation of the reproductive behavior of *Z. tau* and the associated gene expression pattern remains unknown. Additionally, increased ambient temperatures are generally accompanied by changes in carbon dioxide and humidity (Piotr et al., 2017). Therefore, evaluating the response and the underlying molecular mechanism in *Z. tau* individuals under the action of multiple factors is necessary to formulate novel strategies and approaches for dealing with such circumstances.

## Data Availability

The original contributions presented in the study are included in the article/supplementary material, further inquiries can be directed to the corresponding author.
